# Supplementary material for: Activated Carbon with Ultrahigh Specific Surface Derived from Bamboo Shoot Shell through K2FeO4 Oxidative Pyrolysis for Adsorption of Methylene Blue
Source: Molecules. 2023 Apr 12;28(8):3410. doi: 10.3390/molecules28083410 (PMC10145064; doi:10.3390/molecules28083410)
Supplement: Supplementary file 1 [file molecules-28-03410-s001.zip › molecules-2291156-supplementary.pdf]

Supplementary data

Activated carbon with ultrahigh specific surface derived by bamboo shoot shells through K_2FeO_4 oxidative pyrolysis for adsorption of cationic dyes

Text S1 Characterization

Text S2 The kinetic and isotherm models

Text S1 Characterization

The morphology and structure of BACs were characterized through a Scanning electron microscope (SEM, GeminiSEM360) and an X-ray powder diffractometer (XRD, X PERTPRO-30X). The chemical properties on the surface of BACs were investigated by an Axis Ultra X-ray photoelectron spectrometer (XPS, Thermo) and a Fourier transform infrared spectrometer (FTIR, Nexus 670). N_2 adsorption/desorption isotherms were acquired on a BSD-PMR2 surface area and porosity analyzer (BeiShiDe) at 77 K to evaluate the pore structure of BACs. Brunauer-Emmett-Teller (BET) method and non-local density functional theory (NLDFT) model were used to calculate the specific surface area (SSA) and pore size distribution of the BACs.

Text S2 The kinetic and isotherm models

The isotherm data was described by Langmuir (*Eq* (1-2)), Freundlich (*Eq* (3)), Temkin (*Eq* (4)) and Dubinin–Radushkevich (*Eq* (5-7)) models.

$$\frac{q_e}{C_e} = \frac{1}{K_L q_m} + \frac{C_e}{q_m} \tag{1}$$

$$R_L = \frac{1}{1+K_L C_0} \tag{2}$$

$$lnq_e = lnK_F + \frac{1}{n} lnC_e \tag{3}$$

$$q_e = BlnK_T + BlnC_e \tag{4}$$

$$lnq_e = lnq_m - k\varepsilon^2 \tag{5}$$

$$\varepsilon = \mathrm{RT} ln(1+1/C_e) \tag{6}$$

$$\mathrm{E} = 1/\sqrt{2\mathrm{k}} \tag{7}$$

where q_e (mg/g) was the adsorption capacity of adsorbent at equilibrium. C_e was the equilibrium concentrations of MB (mg/L), q_m was the maximum adsorption

capacity (mg/g), K_L is the Langmuir constant related to the rate of adsorption (L/mg), which can be calculated from the plot C_e/q_e vs. C_e. R_L value illustrated the adsorption process was unfavorable (R_L>1) or favorable (0<R_L<1). KF is the Freundlich constant and 1/n is the heterogeneity factor, which can be obtained from the plot lnq_e vs. lnC_e. *1/n* reflected the isotherms type, 0<1/*n*<1, it was desirable, while 1/*n*>1, it was undesirable. K_T is the equilibrium binding constant, which can be calculated from the plot q_e vs. lnC_e (mg/L). R is the general gas constant (8.314 J/mol/ K), T is absolute temperature (K).

Pseudo-first-order (*Eq* (8)), pseudo-second-order (*Eq* (9)) and intraparticle diffusion (*Eq* (10)) models were used to fit the kinetic data of MB adsorption onto BAC.

$$\frac{d_q}{d_t} = k_1(q_e-q) \quad (8)$$

$$\frac{t}{q_t} = \frac{1}{k_2 q_e^2} + \frac{t}{q_e} \quad (9)$$

$$q_t = k_i t^{1/2} + C \quad (10)$$

Where k_1 (min^{-1}), k_2 ($g\ mg^{-1}\ min^{-1}$) and K_i ($mg\ g^{-1}\ min^{1/2}$) were the constants of pseudo-first-order, pseudo-second-order and intraparticle diffusion model, respectively. C reflected the thickness of boundary layer and the higher C value indicated that its greater influence on adsorption rate.
